# Social cycling and conditional responses in the Rock-Paper-Scissors game

**DOI:** 10.1038/srep05830

**Published:** 2014-07-25

**Authors:** Zhijian Wang, Bin Xu, Hai-Jun Zhou

**Affiliations:** 1 Experimental Social Science Laboratory, Zhejiang University, Hangzhou 310058, China; 2 Public Administration College, Zhejiang Gongshang University, Hangzhou 310018, China; 3 State Key Laboratory of Theoretical Physics, Institute of Theoretical Physics, Chinese Academy of Sciences, Beijing 100190, China; 4 These authors contributed equally to this work.

## Abstract

How humans make decisions in non-cooperative strategic interactions is a big question. For the fundamental Rock-Paper-Scissors (RPS) model game system, classic Nash equilibrium (NE) theory predicts that players randomize completely their action choices to avoid being exploited, while evolutionary game theory of bounded rationality in general predicts persistent cyclic motions, especially in finite populations. However as empirical studies have been relatively sparse, it is still a controversial issue as to which theoretical framework is more appropriate to describe decision-making of human subjects. Here we observe population-level persistent cyclic motions in a laboratory experiment of the discrete-time iterated RPS game under the traditional random pairwise-matching protocol. This collective behavior contradicts with the NE theory but is quantitatively explained, without any adjustable parameter, by a microscopic model of win-lose-tie conditional response. Theoretical calculations suggest that if all players adopt the same optimized conditional response strategy, their accumulated payoff will be much higher than the reference value of the NE mixed strategy. Our work demonstrates the feasibility of understanding human competition behaviors from the angle of non-equilibrium statistical physics.

The Rock-Paper-Scissors (RPS) game is a fundamental non-cooperative game. It has been widely used to study competition phenomena in society and biology, such as species diversity of ecosystems^1^,^2^,^3^,^4^,^5^,^6^ and price dispersion of markets^7^,^8^. This game has three candidate actions *R* (rock), *P* (paper) and *S* (scissors). In the simplest settings the payoff matrix is characterized by a single parameter, the payoff *a* of the winning action (*a* > 1, see Fig. 1A)^9^. There are the following non-transitive dominance relations among the actions: *R* wins over *S*, *P* wins over *R*, yet *S* wins over *P* (Fig. 1B), so no action is absolutely better than the others.

The RPS game is also a basic model system for studying decision-making of human subjects in competitive environments and the associated social dynamics and non-equilibrium physics. Assuming ideal rationality for players who repeatedly playing the RPS game within a population, classical game theory predicts that individual players will completely randomize their action choices so that their behaviors will be unpredictable and not be exploited by the other players^10^,^11^. This is referred to as the mixed-strategy Nash equilibrium (NE), in which every player chooses the three actions with equal probability 1/3 at each game round (see Supplementary Notes online). When the payoff parameter *a* < 2 this NE is evolutionarily unstable with respect to small perturbations but it becomes evolutionarily stable at *a* > 2 (see Supplementary Notes online)^12^. On the other hand, evolutionary game theory drops the infinite rationality assumption and looks at the RPS game from the angle of evolution and adaption^13^,^14^,^15^,^16^,^17^,^18^. Evolutionary models based on various microscopic learning rules (such as the replicator dynamics^12^,^19^,^20^,^21^, the best response dynamics^22^,^23^ and the logit dynamics^24^,^25^) generally predict cyclic evolution patterns for the action marginal distribution (mixed strategy) of each player, especially in finite populations.

Empirical verification of non-equilibrial persistent cycling in the human-subject RPS game (and other non-cooperative games) has been rather nontrivial, as the recorded evolutionary trajectories are usually highly stochastic and not long enough to draw convincing conclusions. Two of the present authors partially overcame these difficulties by using social state velocity vectors^26^ and forward and backward transition vectors^27^ to visualize violation of detailed balance in game evolution trajectories, but a simple way of quantitatively measuring persistent cyclic behavoiors in a highly stochastic trajectory was still lacking. The cycling frequency of directional flows in the neutral RPS game (*a* = 2) was later quantitatively measured in^28^ using a coarse-grained counting technique. Cason and co-workers^29^ using another cycle rotation index as the order parameter also obtained evidence of persistent cycling in some evolutionarily stable RPS-like games, if players were allowed to update actions asynchronously in continuous time and were informed about the social states of the whole population by some sophisticated ‘heat maps'.

In this work we investigate whether cycling is a general aspect even for the simplest RPS game. We adopt an improved cycle counting method on the basis of our earlier experiences^28^ and study directional flows in evolutionarily stable (*a* > 2) and unstable (*a* < 2) discrete-time RPS games. We show strong evidence that the RPS game is an intrinsic non-equilibrium system, which cannot be fully described by the NE concept even in the evolutionarily stable region but rather exhibits persistent population-level cyclic motions. We then bridge the collective cycling behavior and the highly stochastic decision-making of individuals through a simple conditional response (CR) mechanism. Our empirical data confirm the plausibility of this microscopic model of bounded rationality. Our theoretical calculations also demonstrate that, if all the players adopt the same CR strategy and if the transition parameters of this strategy are chosen in an optimized way, this CR strategy will outperform the NE mixed strategy in terms of the accumulated payoffs of individual players, yet the action marginal distribution of individual players is indistinguishable from that of the NE mixed strategy. Our work as a successful attempt of understanding competition dynamics from the perspective of non-equilibrium statistical physics may stimulate future more refined experimental and theoretical studies on the microscopic mechanisms of decision-making and learning in basic game systems^19^,^30^,^31^,^32^,^33^,^34^.

## Results

### Experimental system

We recruited a total number of 360 students from different disciplines of Zhejiang University to form 60 disjoint populations of size *N* = 6. Each population then carries out one experimental session by playing the RPS game 300 rounds (taking 90–150 minutes) with a fixed value of *a*. In real-world situations individuals often have to make decisions based only on partial input information. We mimic such situations by adopting the traditional random pairwise-matching experimental protocol^11^: At each game round (time) *t* the players are randomly paired within the population and compete with their pair opponent once; after that each player gets feedback information about her own payoff as well as her and her opponent's action. As the experimental session finishes, the players are paid in real cash proportional to their accumulated payoffs (see Methods). Our experimental setting differs from those of two other recent experiments, in which every player competes against the whole population^9^,^29^ and may change actions in continuous time^29^. We set *a* = 1.1, 2, 4, 9 and 100, respectively, in one-fifth of the populations so as to compare the dynamical behaviors in the evolutionarily unstable, neutral, stable and deeply stable regions.

### Action marginal distribution of individual players

**Figure 1:**
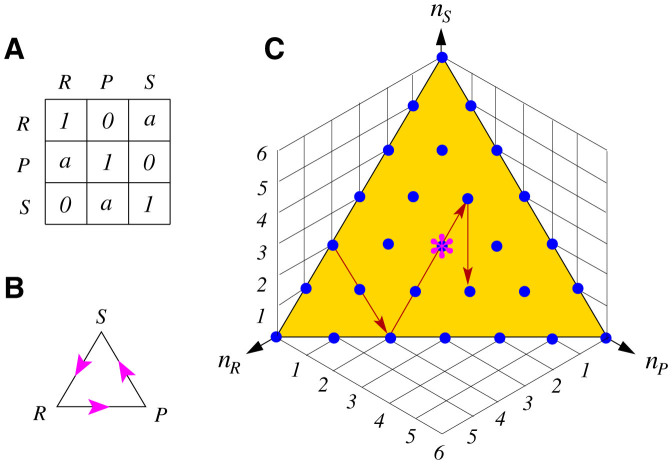
The Rock-Paper-Scissors game. (A) Each matrix entry specifies the row action's payoff. (B) Non-transitive dominance relations (*R* beats *S*, *P* beats *R*, *S* beats *P*) among the three actions. (C) The social state plane for a population of size *N* = 6. Each filled circle denotes a social state (*n_R_*, *n_P_*, *n_S_*); the star marks the centroid **c**_0_; the arrows indicate three social state transitions at game rounds *t* = 1, 2, 3.

**Figure 2:**
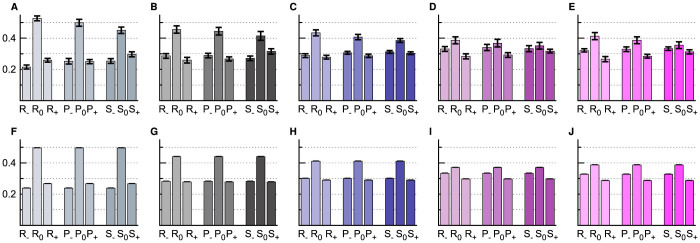
Action shift probability conditional on a player's current action. If a player adopts action *R* at one game round, this player's probability of repeating the same action at the next game round is denoted as *R*_0_, while the probability of performing a counter-clockwise or clockwise action shift is denoted, respectively, as *R*_+_ and *R*_−_. The conditional probabilities *P*_0_, *P*_+_, *P*_−_ and *S*_0_, *S*_+_, *S*_−_ are defined similarly. (A–E) The mean (vertical bin) and the SEM (standard error of the mean, error bar) of each conditional probability obtained by averaging over the different populations of the same payoff parameter *a* = 1.1, 2, 4, 9, and 100 (from left to right). (F–J) The corresponding action shift probability values predicted by the conditional response model using the parameters of Fig. 3F–3J as inputs.

We observe that the individual players shift their actions frequently in all the populations except one with *a* = 1.1 (this exceptional population is discarded from further analysis, see Supplementary Notes online). Averaged among the 354 players of these 59 populations, the probabilities that a player adopts action *R*, *P*, *S* at one game round are, respectively, 0.36 ± 0.08, 0.33 ± 0.07 and 0.32 ± 0.06 (mean ± s.d.). We obtain very similar results for each set of populations of the same *a* value (see Supplementary Table S1 online). These results are consistent with NE and suggest the NE mixed strategy is a good description of a player's marginal distribution of actions. However, a player's actions at two consecutive times are not independent but correlated. As demonstrated in Fig. 2A–2E, at each time the players are more likely to repeat their last action than to shift action either counter-clockwise (i.e., *R* → *P*, *P* → *S*, *S* → *R*, see Fig. 1B) or clockwise (*R* → *S*, *S* → *P*, *P* → *R*). This inertial effect is especially strong at *a* = 1.1 and it diminishes as *a* increases.

We notice that at *a* ≥ 2, an individual player's probability of making a clockwise action shift is equal to or just slightly different from that of making a counter-clockwise action shift (Fig. 2A–2E). There is no or only very weak cycling behavior at the level of individual players in the evolutionarily neutral (*a* = 2) and stable (*a* > 2) RPS games, in accordance with the NE theory. As shown in Fig. 2F–2J, the action shift statistics of individual players can be well explained by the later introduced conditional response model.

### Collective behaviors of the whole population

The social state of the population at any time *t* is denoted as **s**(*t*) ≡ (*n_R_*(*t*), *n_P_*(*t*), *n_S_*(*t*)) with *n_q_* being the number of players adopting action *q* ∈ {*R*, *P*, *S*}. Since *n_R_* + *n_P_* + *n_S_* ≡ *N* there are (*N* + 1)(*N* + 2)/2 such social states, all lying on a three-dimensional plane bounded by an equilateral triangle (Fig. 1C). Each population leaves a trajectory on this plane as the RPS game proceeds. To detect rotational flows, we assign for every social state transition **s**(*t*) → **s**(*t* + 1) a rotation angle *θ*(*t*), which measures the angle this transition rotates with respect to the centroid **c**_0_ ≡ (*N*/3, *N*/3, *N*/3) of the social state plane (see Methods)^28^. Positive and negative *θ* values signify counter-clockwise and clockwise rotations, respectively, while *θ* = 0 means the transition is not a rotation around **c**_0_. For example, we have *θ*(1) = *π*/3, *θ*(2) = 0, and *θ*(3) = −2*π*/3 for the three transitions shown in Fig. 1C.

**Figure 3:**
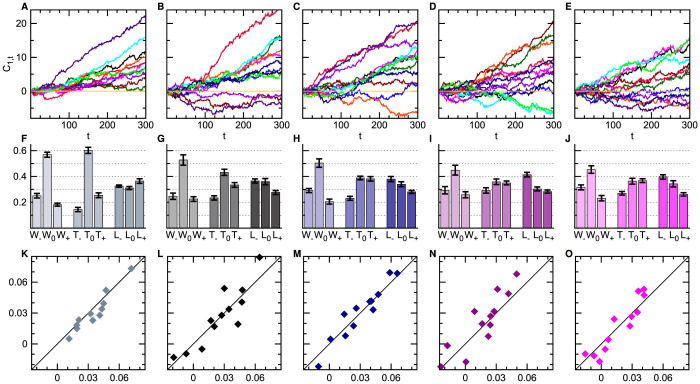
Social cycling explained by conditional response. The payoff parameter is *a* = 1.1, 2, 4, 9 and 100 from left-most column to right-most column. (A–E) Accumulated cycle numbers *C*_1,*t*_ of 59 populations. (F–J) Empirically determined CR parameters, with the mean (vertical bin) and the SEM (error bar) of each CR parameter obtained by considering all the populations of the same *a* value. (K–O) Comparison between the empirical cycling frequency (vertical axis) of each population and the theoretical frequency (horizontal axis) obtained by using the empirical CR parameters of this population as inputs.

The net number of cycles around **c**_0_ during the time interval [*t*_0_, *t*_1_] is computed by 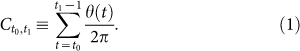
As shown in Fig. 3A–3E, *C*_1,*t*_ has an increasing trend in most of the 59 populations, indicating persistent counter-clockwise cycling. The cycling frequency of each trajectory in [*t*_0_, *t*_1_] is evaluated by 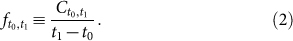
The values of *f*_1,300_ for all the 59 populations are listed in Table 1, from which we obtain the mean frequency to be 0.031 ± 0.006 (*a* = 1.1, mean ± SEM), 0.027 ± 0.008 (*a* = 2), 0.031 ± 0.008 (*a* = 4), 0.022 ± 0.008 (*a* = 9) and 0.018 ± 0.007 (*a* = 100). These mean frequencies are all positive irrespective to the particular value of *a*, indicating that behind the seemingly highly irregular social state evolution process, there is a deterministic pattern of social state cycling from slightly rich in action *R*, to slightly rich in *P*, then to slightly rich in *S*, and then back to slightly rich in *R* again. Statistical analysis confirms that *f*_1,300_ > 0 is significant for all the five sets of populations (Wilcoxon signed-rank test, *p* < 0.05). The correlation between the mean cycling frequency *f*_1,300_ and the payoff parameter *a* is not statistically significant (Spearman's correlation test: *r* = −0.82, *p* = 0.19, for *n* = 5 mean frequencies; and *r* = −0.16, *p* = 0.24, for *n* = 59 frequencies). We also notice that the mean cycling frequency in the second half of the game (*f*_151,300_) is slightly higher than that in the first half (*f*_1,150_) for all the five sets of populations (Supplementary Table S2 online), suggesting that cycling does not die out with time.

A recent experimental work^35^ also observed cycling behaviors in a RPS-like game with more than three actions. Evidences of persistent cycling in some complete-information and continuous-time RPS-like games were reported in another experimental study^29^. However, no (or only very weak) evidence of population-level cycling was detected in^29^ if action updating was performed in discrete time. Here and in Ref. 28 we find that even discrete-time updating of actions will lead to collective cyclic motions in the RPS game, and such a population-level behavior is not affected by the particular value of *a*.

### Empirical conditional response patterns

Under the assumption of mixed-strategy NE (i.e., each player chooses the three actions with equal probability at every game round, independent of each other and of the payoffs of previous plays), the social state transitions should obey the detailed balance condition. Therefore the observed persistent cycling behavior cannot be understood within the NE framework. Persistent cycling can also not be explained by the independent decision model which assumes the action choice of a player at one time is influenced only by her action at the previous time (see Supplementary Notes online). Using the empirically determined action shift probabilities of Fig. 2A–2E as inputs, we find that this independent decision model predicts the cycling frequency to be 0.0050 (for *a* = 1.1), −0.0005 (*a* = 2), −0.0024 (*a* = 4), −0.0075 (*a* = 9) and −0.0081 (*a* = 100), which are all very close to zero and significantly different from the empirical values.

The action choices of different players must be mutually influenced. Our empirical data shown in Fig. 3F–3J confirm the existence of such mutual influences. Let us denote by *O* the performance (output) of a player at a given game round, with *O* ∈ {*W* (win), *T* (tie), *L* (lose)}. Conditional on the output *O*, the probability that this player will decide to shift action clockwise or counter-clockwise or keep the same action in the next play is denoted as *O*_−_, *O*_+_ and *O*_0_ (≡ 1 − *O*_−_ − *O*_+_), respectively. Most interestingly, we see from Fig. 3F–3J that if a player wins over her opponent in one play, her probability (*W*_0_) of repeating the same action in the next play is considerably higher than her probabilities (*W*_−_ and *W*_+_) of shifting actions. Furthermore, for payoff parameter *a* ≥ 2, if a player loses to her opponent in one play, she is more likely to shift action clockwise (probability *L*_−_) than either to keep the old action (*L*_0_) or to shift action counter-clockwise (*L*_+_).

### The conditional response model

Inspired by these empirical observations, we develop a simplest nontrival model by assuming the following conditional response strategy: at each game round, every player review her previous performance *O* ∈ {*W*, *T*, *L*} and makes an action choice according to the corresponding three conditional probabilities (*O*_−_, *O*_0_, *O*_+_). This model is characterized by a set Γ ≡ {*W*_−_, *W*_+_; *T*_−_, *T*_+_; *L*_−_, *L*_+_} of six CR parameters. Notice this CR model differs qualitatively from the discrete-time logit dynamics model^24^,^25^ used in Ref. 28, which assumes each player has global information about the population's social state.

We can solve this win-lose-tie CR model analytically and numerically (see Supplementary Notes online). Let us denote by *n_rr_*, *n_pp_*, *n_ss_*, *n_rp_*, *n_ps_* and *n_sr_*, respectively, as the number of pairs in which the competition being *R*–*R*, *P*–*P*, *S*–*S*, *R*–*P*, *P*–*S*, and *S*–*R*, in one game round *t*. Given the social state **s** = (*n_R_*, *n_P_*, *n_S_*) at time *t*, the conditional joint probability distribution of these six integers is expressed as 
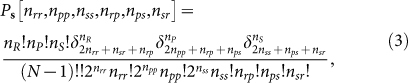
where (*N* − 1)!! ≡ 1 × 3 × … × (*N* − 3) × (*N* − 1) and 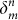 is the Kronecker symbol (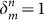 if *m* = *n* and = 0 if otherwise). With the help of this expression, we can then obtain an explicit formula for the social state transition probability *M_cr_*[**s**′|**s**] from **s** to any another social state **s**′ (see Methods). We then compute numerically the steady-state social state distribution 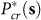 of this Markov matrix^36^ and other average quantities of interest. For example, the mean steady-state cycling frequency *f_cr_* of this model is computed by 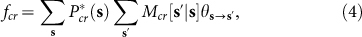
where *θ***_s_**_→**s**′_ is the rotation angle associated with the social state transition **s** → **s**′, see Eq. (7).

Using the empirically determined response parameters as inputs, the CR model predicts the mean cycling frequencies for the five sets of populations to be *f_cr_* = 0.035 (*a* = 1.1), 0.026 (*a* = 2), 0.030 (*a* = 4), 0.018 (*a* = 9) and 0.017 (*a* = 100), agreeing well with the empirical measurements. Such good agreements between model and experiment are achieved also for the 59 individual populations (Fig. 3K–3O). In addition, we find the empirically observed inertial effect of Fig. 2A–2E is quantitatively reproduced by the CR model without any fitting parameter (see Fig. 2F–2J).

**Figure 4:**
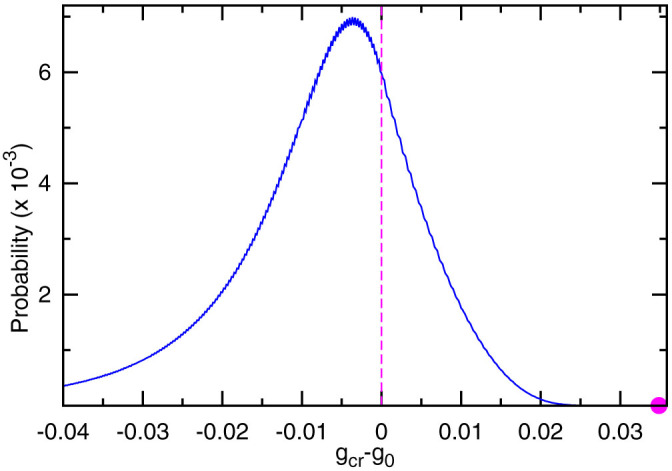
Probability distribution of payoff difference *g_cr_* − *g*_0_ at population size *N* = 6. We assume *a* > 2 and set the unit of the horizontal axis to be (*a* − 2). The solid line is obtained by sampling 2.4 × 10^9^ CR strategies uniformly at random; the filled circle denotes the maximal value of *g_cr_* among these samples.

Because of the rotational symmetry of the conditional response parameters, the CR model predicts that each player's action marginal distribution is uniform, identical to the NE mixed strategy (Supplementary Notes online). On the other hand, according to this model, the expected payoff *g_cr_* per game round of each player is 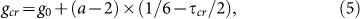
where *g*_0_ ≡ (1 + *a*)/3 is the expected payoff of the NE mixed strategy, and *τ_cr_* is the average fraction of ties among the *N*/2 pairs at each game round, with the expression 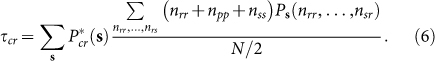
The value of *g_cr_* depends on the CR parameters. By uniformly sampling 2.4 × 10^9^ instances of Γ from the three-dimensional probability simplex, we find that for *a* > 2, *g_cr_* has high chance of being lower than *g*_0_ (Fig. 4), with the mean value of (*g_cr_*−*g*_0_) being −0.0085(*a*−2). (Qualitatively the same conclusion is obtained for larger *N* values, e.g., see Supplementary Fig. S1 online for *N* = 12.) This is consistent with the mixed-strategy NE being evolutionarily stable^12^. On the other hand, the four *g_cr_* values (for the four cases of *a* ≠ 2) determined by the empirical CR parameters and the corresponding four mean payoffs of the empirical data sets all weakly exceed *g*_0_, indicating that individual players are adjusting their responses to achieve higher accumulated payoffs (Supplementary Notes online). The positive gap between *g_cr_* and *g*_0_ may further enlarge if the individual players were given more learning time to optimize their response parameters (e.g., through increasing the repeats of the game).

As shown in Fig. 4 and Supplementary Fig. S1 online, the CR parameters have to be highly optimized to achieve a large value of *g_cr_*. For population size *N* = 6 we give three examples of the sampled best CR strategies for *a* > 2: Γ_1_ = {0.002, 0.000; 0.067, 0.110; 0.003, 0.003}, with cycling frequency *f_cr_* = 0.003 and *g_cr_* = *g*_0_ + 0.035(*a* − 2); Γ_2_ = {0.995, 0.001; 0.800, 0.058; 0.988, 0.012}, with *f_cr_* = −0.190 and *g_cr_* = *g*_0_ + 0.034(*a* − 2); Γ_3_ = {0.001, 0.004; 0.063, 0.791; 0.989, 0.001}, with *f_cr_* = 0.189 and *g_cr_* = *g*_0_ + 0.033(*a* − 2). For large *a* these CR strategies outperform the NE mixed strategy in payoff by about 10%. Set Γ_1_ indicates that population-level cycling is not a necessary condition for achieving high payoff values. On the other hand, set Γ_3_ implies *W*_0_ ≈ 1, *L*_0_ ≈ 0, therefore this CR strategy can be regarded as an extension of the win-stay lose-shift (also called Pavlov) strategy, which has been shown by computer simulations to facilitate cooperation in the prisoner's dilemma game^37^,^38^,^39^,^40^. We should also emphasize that the empirically observed CR transition parameters (Fig. 3F–3J) still differ considerably from those of the win-stay lose-shift strategy Γ_3_.

## Discussion

In game-theory literature it is common to equate individual players' action marginal distributions with their actual strategies^11^,^18^. In reality, however, decision-making and learning are very complicated neural processes^41^,^42^,^43^,^44^,^45^. The action marginal distributions are only a consequence of such complex dynamical processes, their coarse-grained nature makes them unsuitable to describe dynamical properties^17^. Our work on the finite-population RPS game clearly demonstrates this point. This game exhibits persistent cyclic motions at the population level (but not at the individual player level) which cannot be understood by the NE concept but are successfully explained by the empirical data-inspired CR mechanism. As far as the action marginal distributions of individual players are concerned, the CR strategy is indistinguishable from the NE mixed strategy, yet it is capable of bringing higher payoffs to the players if its parameters are optimized and all players adopt the same CR strategy. This simple conditional response strategy, with the win-stay lose-shift strategy being a special case, appears to be psychologically plausible for human subjects with bounded rationality^46^,^47^. For more complicated game payoff matrices, we can generalize the conditional response model accordingly by introducing a larger set of CR parameters (see Supplementary Notes online). It should be very interesting to re-analyze many existing laboratory experimental data^9^,^29^,^35^,^48^,^49^,^50^,^51^ using this extended model. Figure 3 also reveals that the empirical CR parameters and the social-state cycling frequency change with the payoff parameter *a*. In a following paper we will study the effect of the payoff parameter *a* to the individual- and population-level behaviors in more detail^52^.

The CR model as a simple model of decision-making under uncertainty deserves to be fully explored. For example, different players may have different CR transition parameters and these transition parameters may change with time constantly as a result of learning. We find the cycling frequency is not sensitive to population size *N* at given CR parameters (see Supplementary Fig. S2 online); and the cycling frequency is nonzero even for symmetric CR parameters (i.e., *W*_+_/*W*_−_ = *T*_+_/*T*_−_ = *L*_+_/*L*_−_ = 1), as long as *W*_0_ ≠ *L*_0_ (see Supplementary Fig. S3 online). The optimization issue of CR parameters is left out in this work. We will investigate whether an optimal CR strategy is achievable through simple stochastic learning rules^42^,^43^,^45^. The effects of memory length^53^ and population size to the optimal CR strategies also need to be thoroughly studied. On the more biological side, whether conditional response is a basic decision-making mechanism of the human brain or just a consequence of more fundamental neural mechanisms is a challenging question for future studies.

## Methods

### Experiment

The experiment was approved by the Experimental Social Science Laboratory of Zhejiang University and performed at Zhejiang University in the period of December 2010 to March 2014. The corresponding author confirms that this experiment was performed in accordance with the approved social experiments guidelines and regulations. A total number of 360 undergraduate and graduate students of Zhejiang University volunteered to serve as the human subjects of this experiment. These students were openly recruited through a web registration system. Female students were slightly more enthusiastic than male students in registering as candidate human subjects of our experiment. Since we sampled students uniformly at random from the candidate list, more female students were recruited than male students (among the 360 students, the female versus male ratio is 217:143). Informed consent was obtained from all the participanting human subjects.

The 360 human subjects (referred to as players in this work) were distributed into 60 populations of equal size *N* = 6. The six players of each population carried one experimental session by playing the RPS game for 300 rounds with fixed payoff parameter *a*, whose value is chosen from {1.1, 2, 4, 9, 100}. During the game process the players sited separately in a classroom, each of which facing a computer screen. They were not allowed to communicate with each other during the whole experimental session. Written instructions were handed out to each player and the rules of the experiment were also orally explained by an experimental instructor. The rules of the experimental session are as follows:

- Each player plays the RPS game repeatedly with the same other five players.
- Each player earns virtual points during the experimental session according to the payoff matrix shown in the written instruction. These virtual points are then exchanged into RMB as a reward to the player, plus an additional 5 RMB as show-up fee.
- In each game round, the six players of each group are randomly matched by a computer program to form three pairs, and each player competes only with the pair opponent.
- Each player has at most 40 seconds in one game round to make a choice among the three candidate actions “Rock”, “Paper” and “Scissors”. If this time runs out, the player has to make a choice immediately (the experimental instructor will loudly urge these players to do so). After a choice has been made it can not be changed.

Before the start of the actual experimental session, the player were asked to answer four questions to ensure that they understand completely the rules of the experimental session. These four questions are: (1) *If you choose “Rock” and your opponent chooses “Scissors”, how many virtual points will you earn?* (2) *If you choose “Rock” and your opponent chooses also “Rock”, how many virtual points will you earn?* (3) *If you choose “Scissors” and your opponent chooses “Rock”, how many virtual points will you earn?* (4) *Do you know that at each game round you will play with a randomly chosen opponent from your group (yes/no)?*

During the experimental session, the computer screen of each player will show an information window and a decision window. The window on the left of the computer screen is the information window. The upper panel of this information window shows the current game round, the time limit (40 seconds) of making a choice, and the time left to make a choice. The color of this upper panel turns to green at the start of each game round. The color will change to yellow if the player does not make a choice within 20 seconds. The color will change to red if the decision time runs out (and then the experimental instructor will loudly urge the players to make a choice immediately). The color will change to blue if a choice has been made by the player. After all the players of the group have made their decisions, the lower panel of the information window will show the player's own choice, the opponent's choice, and the player's own payoff in this game round. The player's own accumulated payoff is also shown. The players are asked to record their choices of each round on the record sheet (Rock as *R*, Paper as *P*, and Scissors as *S*).

The window on the right of the computer screen is the decision window. It is activated only after all the players of the group have made their choices. The upper panel of this decision window lists the current game round, while the lower panel lists the three candidate actions “Rock”, “Scissors”, “Paper” horizontally from left to right. The player can make a choice by clicking on the corresponding action names. After a choice has been made by the player, the decision window becomes inactive until the next game round starts.

The reward in RMB for each player is determined by the following formula. Suppose a player *i* earns *x_i_* virtual points in the whole experimental session, the total reward *y_i_* in RMB for this player is then given by 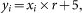
where *r* is the exchange rate between virtual point and RMB. According to the mixed-strategy Nash equilibrium, the expected payoff of each player in one game round is (1 + *a*)/3. Therefore we set the exchange rate to be *r* = 0.45/(1 + *a*) to ensure that, under the mixed-strategy NE assumption, the expected total earning in RMB for a player will be 50 RMB irrespective of the particular experimental session. The value of the payoff parameter *a*, the numerical value of *r*, and the above-mentioned reward formula were listed in the written instruction and also orally mentioned by the experimental instructor at the instruction phase of the experiment.

### Rotation angle computation

Consider a transition from one social state **s** = (*n_R_*, *n_P_*, *n_S_*) at game round *t* to another social state 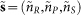 at game round (*t* + 1), if at least one of the two social states coincides with the centroid **c**_0_ of the social state plane, or the three points **s**, 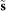 and **c**_0_ lie on a straight line, then the transition 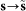 is not regarded as a rotation around **c**_0_, and the rotation angle *θ* = 0. In all the other cases, the transition 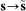 is regarded as a rotation around **c**_0_, and the rotation angle is computed through 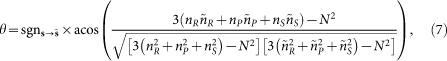
where acos(*x*) ∈ [0, *π*) is the inverse cosine function, and 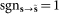 if 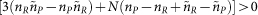 (counter-clockwise rotation around **c**_0_) and 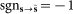 if otherwise (clockwise rotation around **c**_0_).

### Statistical Analysis

Statistical analyses, including Wilcoxon signed-rank test and Spearman's rank correlation test, were performed by using stata 12.0 (Stata, College Station, TX).

### Transition matrix of the conditional response model

For the conditional response model, the transition probability *M_cr_*[**s**′|**s**] from the social state **s** ≡ (*n_R_*, *n_P_*, *n_S_*) at time *t* to the social state 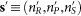 at time (*t* + 1) is expressed as: 
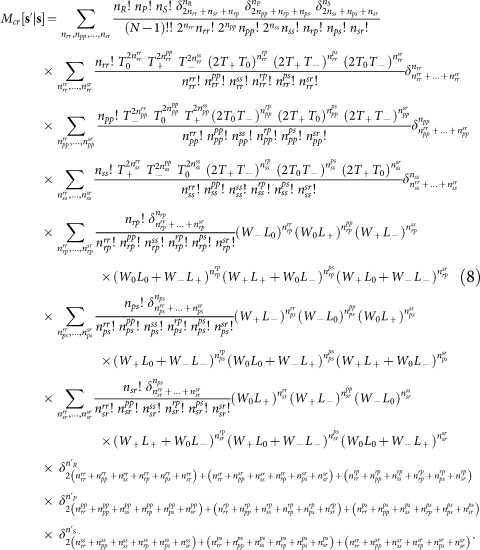


## Supplementary Material

Supplementary Information SIv16Zhou

## Figures and Tables

**Table 1:** Empirical cycling frequencies *f*_1,300_ of 59 populations

	*a* = 1.1	2	4	9	100
	*f*_1,300_	*f*_1,300_	*f*_1,300_	*f*_1,300_	*f*_1,300_
	0.039	0.019	0.033	0.007	0.047
	0.023	0.023	0.005	−0.002	0.004
	0.005	0.054	0.029	0.053	0.024
	0.029	0.034	0.041	0.027	0.051
	0.015	−0.010	0.008	0.068	0.027
	0.052	0.052	0.042	−0.017	0.031
	0.028	0.084	0.069	0.032	0.017
	0.034	0.041	−0.022	0.049	−0.017
	0.073	−0.013	0.069	0.020	−0.012
	0.023	0.017	0.035	−0.022	0.053
	0.018	−0.005	0.048	0.018	−0.010
		0.028	0.018	0.032	−0.005
*µ*	0.031	0.027	0.031	0.022	0.018
*σ*	0.019	0.029	0.026	0.027	0.025
*δ*	0.006	0.008	0.008	0.008	0.007

*µ*: the mean cycling frequency, *σ*: the standard deviation (s.d.) of cycling frequencies, *δ*: the standard error (SEM) of the mean cycling frequency (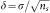, with sample number *n_s_* = 11 for *a* = 1.1 and *n_s_* = 12 for *a* = 2, 4, 9 and 100).
