# Supplementary material for: Social cycling and conditional responses in the Rock-Paper-Scissors game
Source: Sci Rep. 2014 Jul 25;4:5830. doi: 10.1038/srep05830 (PMC5376050; doi:10.1038/srep05830)
Supplement: Supplementary Information — SIv16Zhou [file srep05830-s1.pdf]

Social cycling and conditional responses in the Rock-Paper-Scissors game

Zhijian Wang, Bin Xu, and Hai-Jun Zhou

Supplementary Information

Supplementary Table S1

Supplementary Table S1: Statistics on individual players' action marginal probabilities.

a	m	action	μ	σ	Min	Max
1.1	66	R	0.37	0.08	0.19	0.68
		P	0.34	0.07	0.18	0.52
		S	0.30	0.06	0.09	0.41
2	72	R	0.36	0.07	0.14	0.60
		P	0.32	0.07	0.15	0.58
		S	0.32	0.06	0.13	0.46
4	72	R	0.35	0.08	0.11	0.60
		P	0.33	0.07	0.14	0.54
		S	0.32	0.07	0.11	0.50
9	72	R	0.35	0.08	0.21	0.63
		P	0.33	0.07	0.13	0.55
		S	0.32	0.06	0.16	0.53
100	72	R	0.35	0.07	0.22	0.60
		P	0.33	0.05	0.16	0.51
		S	0.32	0.06	0.14	0.47
	354	R	0.36	0.08	0.11	0.68
		P	0.33	0.07	0.13	0.58
		S	0.32	0.06	0.09	0.53

m is the total number of players; μ, σ, Max and Min are, respectively, the mean, the standard deviation (s.d.), the maximum and minimum of the action marginal probability in question among all the m players. The last three rows are statistics performed on all the 354 players.

Supplementary Table S2

Supplementary Table S2: Empirical cycling frequencies $f_{1,150}$ and $f_{151,300}$ for 59 populations.

	1.1		2		4		9		100	
	$f_{1,150}$	$f_{151,300}$	$f_{1,150}$	$f_{151,300}$	$f_{1,150}$	$f_{151,300}$	$f_{1,150}$	$f_{151,300}$	$f_{1,150}$	$f_{151,300}$
	0.032	0.047	0.020	0.017	0.016	0.050	−0.007	0.022	0.040	0.052
	0.008	0.039	0.028	0.017	−0.002	0.014	−0.005	0.001	−0.003	0.009
	0.025	−0.014	0.021	0.087	0.023	0.035	0.044	0.062	0.009	0.038
	0.015	0.045	0.023	0.043	0.024	0.059	0.034	0.020	0.045	0.060
	0.011	0.019	−0.027	0.006	0.019	−0.004	0.045	0.088	0.017	0.038
	0.036	0.068	0.024	0.081	0.018	0.068	−0.022	−0.014	0.055	0.006
	0.010	0.045	0.083	0.086	0.079	0.059	0.032	0.030	0.008	0.026
	0.036	0.033	0.034	0.046	−0.013	−0.031	0.047	0.050	−0.019	−0.015
	0.076	0.070	−0.032	0.004	0.077	0.061	0.010	0.027	−0.034	0.010
	0.029	0.016	0.034	−0.002	0.018	0.051	−0.003	−0.041	0.055	0.052
	0.009	0.025	−0.004	−0.006	0.061	0.038	0.005	0.029	−0.012	−0.007
			0.022	0.031	0.017	0.019	0.011	0.055	−0.017	0.007
μ	0.026	0.036	0.019	0.034	0.028	0.035	0.016	0.027	0.012	0.023
σ	0.020	0.024	0.030	0.035	0.029	0.030	0.024	0.035	0.031	0.025
δ	0.006	0.007	0.009	0.010	0.008	0.009	0.007	0.010	0.009	0.007

The first row shows the value of the payoff parameter a. For each experimental session (population), $f_{1,150}$ and $f_{151,300}$ are respectively the cycling frequency in the first and the second 150 time steps. μ is the mean cycling frequency, σ is the standard deviation (s.d.) of the cycling frequency, $\delta = \sigma/\sqrt{n_s}$ is the standard error (SEM) of the mean cycling frequency. The number of populations is $n_s = 11$ for $a = 1.1$ and $n_s = 12$ for $a = 2$, 4, 9 and 100.

Supplementary Figure S1

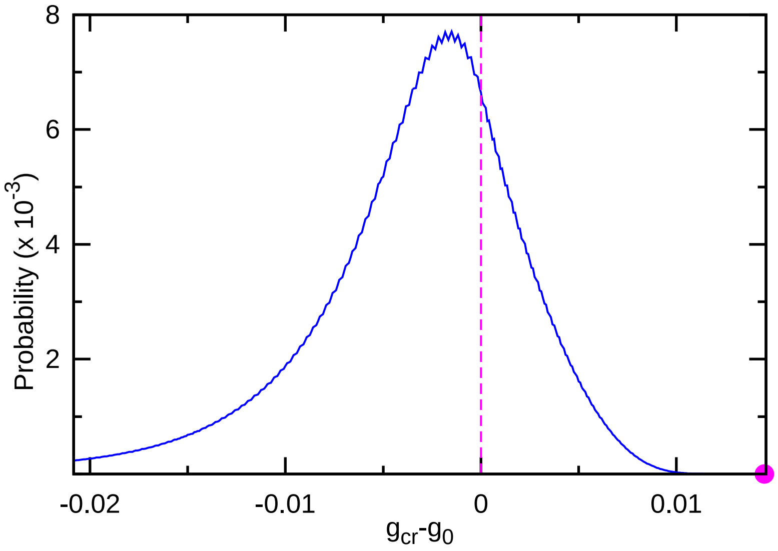

Supplementary Figure S1: Probability distribution of payoff difference $g_{cr} - g_0$ at population size $N = 12$. As in Fig. 4, we assume $a > 2$ and set the unit of the horizontal axis to be $(a - 2)$. The solid line is obtained by sampling 2.4×10^9 CR strategies uniformly at random; the filled circle denotes the maximal value of g_{cr} among these samples.

Supplementary Figure S2

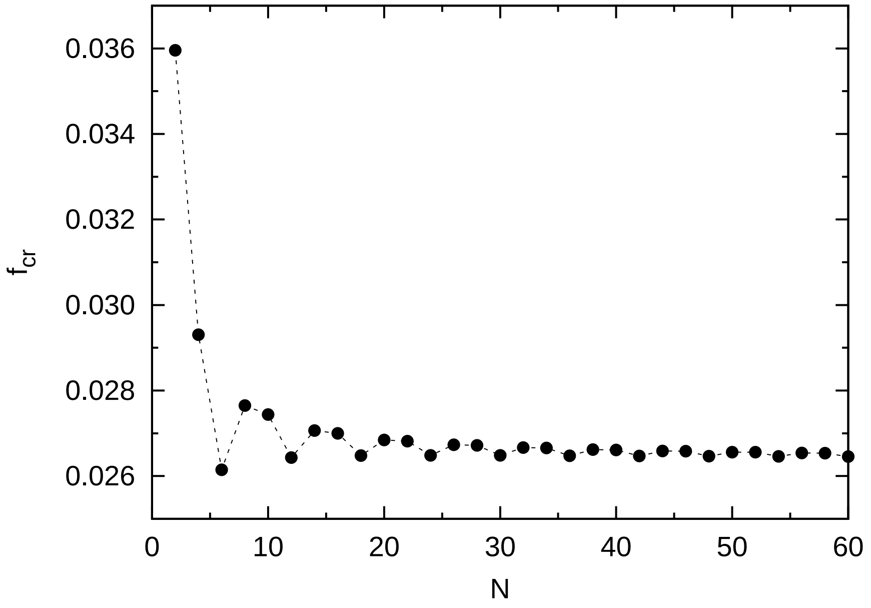

Supplementary Figure S2: The cycling frequency f_{cr} of the conditional response model as a function of population size N. For the purpose of illustration, the CR parameters shown in Fig. 3G are used in the numerical computations.

Supplementary Figure S3

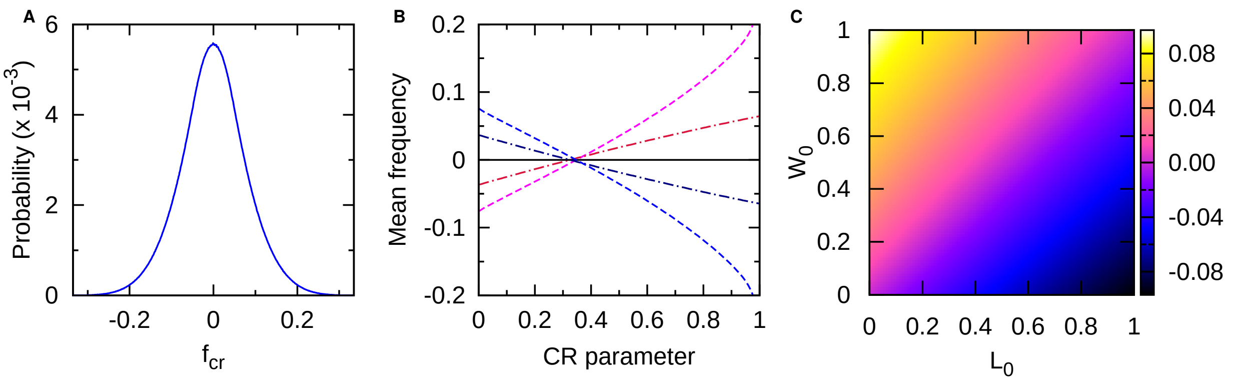

Supplementary Figure S3: Theoretical predictions of the conditional response model with population size $N = 6$. (A) Probability distribution of the cycling frequency f_{cr} obtained by sampling 2.4×10^9 CR strategies uniformly at random. (B) The mean value of f_{cr} as a function of one fixed CR parameter while the remaining CR parameters are sampled uniformly at random. The fixed CR parametr is T_+ (red dashed line), W_0 or L_+ (brown dot-dashed line), W_+ or T_0 or L_- (black solid line), W_- or L_0 (purple dot-dashed line), and T_- (blue dashed line). (C) Cycling frequency f_{cr} as a function of CR parameters W_0 and L_0 for the symmetric CR model ($W_+/W_- = T_+/T_- = L_+/L_- = 1$) with $T_0 = 0.333$.

Supplementary Notes

S1. THE MIXED-STRATEGY NASH EQUILIBRIUM

The RPS game has a mixed-strategy Nash equilibrium (NE), in which every player of the population adopts the three actions (R, P, and S) with the same probability 1/3 in each round of the game. Here we give a proof of this statement. We also demonstrate that, the empirically observed action marginal probabilities of individual players are consistent with the NE mixed strategy.

Consider a population of N individuals playing repeatedly the RPS game under the random pairwise-matching protocol. Let us define ρ_i^R (respectively, ρ_i^P and ρ_i^S) as the probability that a player i of the population ($i \in \{1, 2, \ldots, N\}$) will choose action R (respectively, P and S) in one game round. If a player j chooses action R, what is her expected payoff in one play? Since this player has equal chance $1/(N-1)$ of pairing with any another player i, the expected payoff is simply $g_j^R \equiv \sum_{i \neq j} (\rho_i^R + a\rho_i^S)/(N-1)$. By the same argument we see that if player j chooses action P and S the expected payoff g_j^P and g_j^S in one play are, respectively, $g_j^P \equiv \sum_{i \neq j} (\rho_i^P + a\rho_i^R)/(N-1)$ and $g_j^S \equiv \sum_{i \neq j} (\rho_i^S + a\rho_i^P)/(N-1)$.

If every player of the population chooses the three actions with equal probability, namely that $\rho_i^R = \rho_i^P = \rho_i^S = 1/3$ for $i = 1, 2, \ldots, N$, then the expected payoff for a player is the same no matter which action she chooses in one round of the game, i.e., $g_i^R = g_i^P = g_i^S = (1+a)/3$ for $i = 1, 2, \ldots, N$. Then the expected payoff of a player i in one game round is $(1+a)/3$, which will not increase if the probabilities $\rho_i^R, \rho_i^P, \rho_i^S$ deviate from 1/3. Therefore $\rho_i^R = \rho_i^P = \rho_i^S = 1/3$ (for all $i = 1, 2, \ldots, N$) is a mixed-strategy NE of the game.

Let us also discuss a little bit about the uniqueness of this mixed-strategy NE. If the payoff parameter $a \leq 1$, this mixed-strategy NE is not unique. We can easily check that $\rho_i^R = 1, \rho_i^P = \rho_i^S = 0$ (for all $i = 1, 2, \ldots, N$) is a pure-strategy NE. Similarly, $\rho_i^P = 1, \rho_i^S = \rho_i^R = 0$ ($i = 1, 2, \ldots, N$) and $\rho_i^S = 1, \rho_i^R = \rho_i^P = 0$ ($i = 1, 2, \ldots, N$) are two other pure-strategy NEs. In such a pure-strategy NE the payoff of a player is 1 in one game round. This value is considerably higher than the average payoff of $(1+a)/3$ a player will gain if the population is in the above mentioned mixed-strategy NE.

On the other hand, if the payoff parameter $a > 1$, then there is no pure-strategy NE for the RPS game. This is simple to prove. Suppose the population is initially in a pure-strategy

NE with $\rho_i^R = 1$ for $i = 1, 2, \ldots, N$. If one player now shifts to action S, her payoff will increase from 1 to a. Therefore this player will keep the new action S in later rounds of the game, and the original pure-strategy NE is then destroyed.

We believe that the mixed-strategy NE of $\rho_i^R = \rho_i^P = \rho_i^S = 1/3$ (for $i = 1, 2, \ldots, N$) is the only Nash equilibrium of our RPS game in the whole parameter region of $a > 1$ (except for very few isolated values of a, maybe). Unfortunately we are unable to offer a rigorous proof of this conjecture for a generic value of population size N. But this conjecture is supported by our empirical observations, see Supplementary Table S1 online.

Among the 60 experimental sessions performed at different values of a, we observed that all the players in 59 experimental sessions change their actions frequently. The mean values of the individual action probabilities ρ_i^R, ρ_i^P, ρ_i^S are all close to 1/3. (The slightly higher mean probability of choosing action R in the empirical data of Supplementary Table S1 online might be linked to the fact that "Rock" is the left-most candidate choice in each player's decision window.)

We did notice considerable deviation from the NE mixed strategy in one experimental session of $a = 1.1$, though. After the RPS game has proceeded for 72 rounds, the six players of this exceptional session all stick to the same action R and do not shift to the other two actions. This population obviously has reached a highly cooperative state after 72 game rounds with $\rho_i^R = 1$ for all the six players. As we have pointed out, such a cooperative state is not a pure-strategy NE. We do not consider this exceptional experimental session in the data analysis and model building phase of this work.

S2. EVOLUTIONARY STABILITY OF THE NASH EQUILIBRIUM

We now demonstrate that the mixed-strategy NE with $\rho_i^R = \rho_i^P = \rho_i^S = 1/3$ ($i = 1, 2, \ldots, N$) is an evolutionarily stable strategy only when the payoff parameter $a > 2$.

To check for the evolutionary stability of this mixed-strategy NE, let us assume a mutation occurs to the population such that $n \geq 1$ players now adopt a mutated strategy, while the remaining $(N - n)$ players still adopt the NE mixed strategy. We denote by $\tilde{\rho}^R$ (and respectively, $\tilde{\rho}^P$ and $\tilde{\rho}^S$) as the probability that in one round of the game, action R (respectively, P and S) will be chosen by a player who adopts the mutated strategy. Obviously $\tilde{\rho}^R + \tilde{\rho}^P + \tilde{\rho}^S \equiv 1$.

For a player who adopts the NE mixed strategy, her expected payoff in one game round is simply $g_0 = (1+a)/3$. On the other hand, the expected payoff $\tilde{g}$ in one game round for a player who adopts the mutated strategy is expressed as $\tilde{g} = \tilde{\rho}^R \tilde{g}^R + \tilde{\rho}^P \tilde{g}^P + \tilde{\rho}^S \tilde{g}^S$, where $\tilde{g}^R$ (and respectively, $\tilde{g}^P$ and $\tilde{g}^S$) is the expected payoff of one play for a player in the mutated sub-population if she chooses action R (respectively, P and S):

$$\tilde{g}^R = \frac{N-n}{N-1} \times \frac{1+a}{3} + \frac{n-1}{N-1} \times \left(\tilde{\rho}^R + a\tilde{\rho}^S\right) , \tag{S1}$$

$$\tilde{g}^P = \frac{N-n}{N-1} \times \frac{1+a}{3} + \frac{n-1}{N-1} \times \left(\tilde{\rho}^P + a\tilde{\rho}^R\right) , \tag{S2}$$

$$\tilde{g}^S = \frac{N-n}{N-1} \times \frac{1+a}{3} + \frac{n-1}{N-1} \times \left(\tilde{\rho}^S + a\tilde{\rho}^P\right) . \tag{S3}$$

Inserting these three expressions into the expression of $\tilde{g}$, we obtain that

$$\begin{aligned} \tilde{g} &= \frac{N-n}{N-1} \times \frac{1+a}{3} + \frac{n-1}{N-1} \times \Big(1 + (a-2)\big[\tilde{\rho}^R\tilde{\rho}^P + (\tilde{\rho}^R + \tilde{\rho}^P)(1 - \tilde{\rho}^R - \tilde{\rho}^P)\big]\Big) \\ &= g_0 - \frac{(a-2)(n-1)}{N-1} \times \Big(\big[(\tilde{\rho}^R - 1/3) + (\tilde{\rho}^P/2 - 1/6)\big]^2 + 3\big[\tilde{\rho}^P - 1/3\big]^2/4\Big) . \end{aligned} \tag{S4}$$

If the payoff parameter $a > 2$, we see from Eq. (S4) that the expected payoff $\tilde{g}$ of the mutated strategy never exceeds that of the NE mixed strategy. Therefore the NE mixed strategy is an evolutionarily stable strategy. Notice that the difference $(\tilde{g} - g_0)$ is proportional to $(a-2)$, therefore the larger the value of a, the higher is the cost of deviating from the NE mixed strategy.

On the other hand, in the case of $a < 2$, the value of $\tilde{g} - g_0$ will be positive if two or more players adopt the mutated strategy. Therefore the NE mixed strategy is an evolutionarily unstable strategy.

The mixed-strategy NE for the game with payoff parameter $a = 2$ is referred to as evolutionarily neutral since it is neither evolutionarily stable nor evolutionarily unstable.

S3. CYCLING FREQUENCIES PREDICTED BY TWO SIMPLE MODELS

We now demonstrate that the empirically observed persistent cycling behaviors could not have been observed if the population were in the mixed-strategy NE, and they cannot be explained by the independent decision model either.

A. Assuming the mixed-strategy Nash equilibrium

If the population is in the mixed-strategy NE, each player will pick an action uniformly at random at each time t. Suppose the social state at time t is $\mathbf{s} = (n_R, n_P, n_S)$, then the probability $M_0[\mathbf{s}'|\mathbf{s}]$ of the social state being $\mathbf{s}' = (n'_R, n'_P, n'_S)$ at time $(t+1)$ is simply expressed as

$$M_0[\mathbf{s}'|\mathbf{s}] = \frac{N!}{(n'_R)!(n'_P)!(n'_S)!}\left(\frac{1}{3}\right)^N , \tag{S5}$$

which is independent of $\mathbf{s}$. Because of this history independence, the social state probability distribution $P_0^*(\mathbf{s})$ for any $\mathbf{s} = (n_R, n_P, n_S)$ is

$$P_0^*(\mathbf{s}) = \frac{N!}{n_R! n_P! n_S!}\left(\frac{1}{3}\right)^N . \tag{S6}$$

The social state transition obeys the detailed balance condition that $P_0^*(\mathbf{s})M_0[\mathbf{s}'|\mathbf{s}] = P_0^*(\mathbf{s}')M_0[\mathbf{s}|\mathbf{s}']$. Therefore no persist cycling can exist in the mixed-strategy NE. Starting from any initial social state $\mathbf{s}$, the mean of the social states at the next time step is $\mathbf{d}(\mathbf{s}) = \sum_{\mathbf{s}'} M_0[\mathbf{s}'|\mathbf{s}]\mathbf{s}' = \mathbf{c}_0$, i.e., identical to the centroid of the social state plane.

B. Assuming the independent decision model

In the independent decision model, every player of the population decides on her next action $q' \in \{R, P, S\}$ in a probabilistic manner based on her current action $q \in \{R, P, S\}$ only. For example, if the current action of a player i is R, then in the next game round this player has probability R_0 to repeat action R, probability R_- to shift action clockwise to S, and probability R_+ to shift action counter-clockwise to P. The transition probabilities P_-, P_0, P_+ and S_-, S_0, S_+ are defined in the same way. These nine transition probabilities of cause have to satisfy the normalization conditions: $R_- + R_0 + R_+ = 1$, $P_- + P_0 + P_+ = 1$, and $S_- + S_0 + S_+ = 1$.

Given the social state $\mathbf{s} = (n_R, n_P, n_S)$ at time t, the probability $M_{id}[\mathbf{s}'|\mathbf{s}]$ of the popula-

tion's social state being $\mathbf{s}' = (n'_R, n'_P, n'_S)$ at time $(t+1)$ is

$$
\begin{aligned}
M_{id}[\mathbf{s}'|\mathbf{s}] \;=\; & \sum_{n_{R\to R}} \sum_{n_{R\to P}} \sum_{n_{R\to S}} \frac{n_R!}{n_{R\to R}! n_{R\to P}! n_{R\to S}!} R_-^{n_{R\to S}} R_0^{n_{R\to R}} R_+^{n_{R\to P}} \delta^{n_R}_{n_{R\to R}+n_{R\to P}+n_{R\to S}} \\
& \times \sum_{n_{P\to R}} \sum_{n_{P\to P}} \sum_{n_{P\to S}} \frac{n_P!}{n_{P\to R}! n_{P\to P}! n_{P\to S}!} P_-^{n_{P\to R}} P_0^{n_{P\to P}} P_+^{n_{P\to S}} \delta^{n_P}_{n_{P\to R}+n_{P\to P}+n_{P\to S}} \\
& \times \sum_{n_{S\to R}} \sum_{n_{S\to P}} \sum_{n_{S\to S}} \frac{n_S!}{n_{S\to R}! n_{S\to P}! n_{S\to S}!} S_-^{n_{S\to P}} S_0^{n_{S\to S}} S_+^{n_{S\to R}} \delta^{n_S}_{n_{S\to R}+n_{S\to P}+n_{S\to S}} \\
& \times \delta^{n'_R}_{n_{R\to R}+n_{P\to R}+n_{S\to R}} \delta^{n'_P}_{n_{R\to P}+n_{P\to P}+n_{S\to P}} \delta^{n'_S}_{n_{R\to S}+n_{P\to S}+n_{S\to S}} \; , \qquad \text{(S7)}
\end{aligned}
$$

where $n_{q\to q'}$ denotes the total number of action transitions from q to q', and δ^n_m is the Kronecker symbol such that $\delta^n_m = 1$ if $m = n$ and $\delta^n_m = 0$ if $m \neq n$.

For this independent decision model, the steady-state distribution $P^*_{id}(\mathbf{s})$ of the social states is determined by solving

$$
P^*_{id}(\mathbf{s}) = \sum_{\mathbf{s}'} M_{id}[\mathbf{s}|\mathbf{s}'] P^*_{id}(\mathbf{s}') \; . \qquad \text{(S8)}
$$

When the population has reached this steady-state distribution, the mean cycling frequency f_{id} is then computed as

$$
f_{id} = \sum_{\mathbf{s}} P^*_{id}(\mathbf{s}) \sum_{\mathbf{s}'} M_{id}[\mathbf{s}'|\mathbf{s}] \theta_{\mathbf{s}\to\mathbf{s}'} \; , \qquad \text{(S9)}
$$

where $\theta_{\mathbf{s}\to\mathbf{s}'}$ is the rotation angle associated with the transition $\mathbf{s} \to \mathbf{s}'$, see Eq. (7).

Using the empirically determined action transition probabilities of Fig. 2A-2E as inputs, the independent decision model predicts the cycling frequency to be 0.0050 (for $a = 1.1$), -0.0005 ($a = 2$), -0.0024 ($a = 4$), -0.0075 ($a = 9$) and -0.0081 ($a = 100$), which are all very close to zero and significantly different from the empirical values. Therefore the assumption of players making decisions independently of each other cannot explain population-level cyclic motions.

S4. DETAILS OF THE CONDITIONAL RESPONSE MODEL

A. Social state transition matrix

In the most general case, our win-lose-tie conditional response (CR) model has nine transition parameters, namely W_-, W_0, W_+, T_-, T_0, T_+, L_-, L_0, L_+. These parameters are all non-negative and are constrained by three normalization conditions:

$$W_- + W_0 + W_+ = 1 \ , \quad T_- + T_0 + T_+ = 1 \ , \quad L_- + L_0 + L_+ = 1 \ , \tag{S10}$$

therefore the three vectors (W_-, W_0, W_+), (T_-, T_0, T_+) and (L_-, L_0, L_+) represent three points of the three-dimensional simplex. Because of Eq. (S10), we can use a set $\Gamma \equiv \{W_-, W_+; T_-, T_+; L_-, L_+\}$ of six transition probabilities to denote a conditional response strategy.

The parameters W_+ and W_- are, respectively, the conditional probability that a player (say i) will perform a counter-clockwise or clockwise action shift in the next game round, given that she wins over the opponent (say j) in the current game round. Similarly the parameters T_+ and T_- are the two action shift probabilities conditional on the current play being a tie, while L_+ and L_- are the action shift probabilities conditional on the current play outcome being 'lose'. The parameters W_0, T_0, L_0 are the probabilities of a player repeating the same action in the next play given the current play outcome being 'win', 'tie' and 'lose', respectively. For example, if the current action of i is R and that of j is S, the joint probability of i choosing action P and j choosing action S in the next play is W_+L_0; while if both players choose R in the current play, the joint probability of player i choosing P and player j choosing S in the next play is then T_+T_-.

We denote by $\mathbf{s} \equiv (n_R, n_P, n_S)$ a social state of the population, where n_R, n_P, and n_S are the number of players who adopt action R, P and S in one round of play, respectively. Since $n_R + n_P + n_S \equiv N$ there are $(N+1)(N+2)/2$ such social states, all lying on a three-dimensional plane bounded by an equilateral triangle (Fig. 1C).

Furthermore we denote by n_{rr}, n_{pp}, n_{ss}, n_{rp}, n_{ps} and n_{sr}, respectively, the number of pairs in which the competition being R–R, P–P, S–S, R–P, P–S, and S–R, in this round of play.

These nine integer values are not independent but are related by the following equations:

$$n_R = 2n_{rr} + n_{sr} + n_{rp} \ , \quad n_P = 2n_{pp} + n_{rp} + n_{ps} \ , \quad n_S = 2n_{ss} + n_{ps} + n_{sr} \ . \tag{S11}$$

Knowing the values of n_R, n_P, n_S is not sufficient to uniquely fix the values of $n_{rr}, n_{pp}, \ldots, n_{sr}$. the conditional joint probability distribution of $n_{rr}, n_{pp}, n_{ss}, n_{rp}, n_{ps}, n_{sr}$ is expressed as Eq. (3). To understand this expression, let us first notice that the total number of pairing patterns of N players is equal to

$$\frac{N!}{(N/2)!\ 2^{N/2}} = (N-1)!! \ ,$$

which is independent of the specific values of n_R, n_P, n_S; and second, the number of pairing patterns with n_{rr} R–R pairs, n_{pp} P–P pairs, ..., and n_{sr} S–R pairs is equal to

$$\frac{n_R!\ n_P!\ n_S!}{2^{n_{rr}} n_{rr}!\ 2^{n_{pp}} n_{pp}!\ 2^{n_{ss}} n_{ss}!\ n_{rp}!\ n_{ps}!\ n_{sr}!} \ .$$

Given the values of $n_{rr}, n_{pp}, \ldots, n_{sr}$ which describe the current pairing pattern, the conditional probability of the social state in the next round of play can be determined. We just need to carefully analyze the conditional probability for each player of the population. For example, consider a R–P pair at game round t. This is a lose–win pair, therefore the two involved players will determine their actions of the next game round according to the CR parameters (L_-, L_0, L_+) and (W_-, W_0, W_+), respectively. At time $(t+1)$ there are six possible outcomes: (rr) both players take action R, with probability $L_0 W_-$; (pp) both players take action P, with probability $L_+ W_0$; (ss) both players take action S, with probability $L_- W_+$; (rp) one player takes action R while the other takes action P, with probability $(L_0 W_0 + L_+ W_-)$; (ps) one player takes action P while the other takes action S, with probability $(L_+ W_+ + L_- W_0)$; (sr) one player takes action S and the other takes action R, with probability $(L_- W_- + L_0 W_+)$. Among the n_{rp} R–P pairs of time t, let us assume that after the play, n_{rp}^{rr} of these pairs will outcome (rr), n_{rp}^{pp} of them will outcome (pp), n_{rp}^{ss} of them will outcome (ss), n_{rp}^{rp} of them will outcome (rp), n_{ps}^{ps} of them will outcome (ps), and n_{sr}^{sr} of them will outcome (sr). Similarly we can define a set of non-negative integers to describe the outcome pattern for each of the other five types of pairs.

Under the random pairwise-matching game protocol, our conditional response model

leads to the expression Eq. (8) for the transition probability $M_{cr}[\mathbf{s}'|\mathbf{s}]$ from the social state $\mathbf{s} \equiv (n_R, n_P, n_S)$ at time t to the social state $\mathbf{s}' \equiv (n'_R, n'_P, n'_S)$ at time $(t+1)$.

B. Steady-state properties

It is not easy to further simplify the transition probabilities $M_{cr}[\mathbf{s}'|\mathbf{s}]$, but their values can be determined numerically. Then the steady-state distribution $P^*_{cr}(\mathbf{s})$ of the social states is determined by numerically solving the following equation:

$$P^*_{cr}(\mathbf{s}) = \sum_{\mathbf{s}'} M_{cr}[\mathbf{s}|\mathbf{s}'] P^*_{cr}(\mathbf{s}') \ . \tag{S12}$$

Except for extremely rare cases of the conditional response parameters (e.g., $W_0 = T_0 = L_0 = 1$), the Markov transition matrix defined by Eq. (8) is ergodic, meaning that it is possible to reach from any social state $\mathbf{s}_1$ to any another social state $\mathbf{s}_2$ within a finite number of time steps. This ergodic property guarantees that Eq. (S12) has a unique steady-state solution $P^*(\mathbf{s})$. In the steady-state, the mean cycling frequency f_{cr} of this conditional response model is then computed through Eq. (4) of the main text. And the mean payoff g_{cr} of each player in one game round is obtained by

$$\begin{aligned} g_{cr} &= \frac{1}{N} \sum_{\mathbf{s}} P^*_{cr}(\mathbf{s}) \sum_{n_{rr}, n_{pp}, \ldots, n_{rs}} \mathrm{Prob}_{\mathbf{s}}(n_{rr}, n_{pp}, \ldots, n_{sr}) \\ &\qquad \times \big[2(n_{rr} + n_{pp} + n_{ss}) + a(n_{rp} + n_{ps} + n_{sr}) \big] \\ &= 1 + \frac{(a-2)}{N} \sum_{\mathbf{s}} P^*_{cr}(\mathbf{s}) \sum_{n_{rr}, n_{pp}, \ldots, n_{rs}} \mathrm{Prob}_{\mathbf{s}}(n_{rr}, n_{pp}, \ldots, n_{sr}) [n_{rp} + n_{ps} + n_{sr}] \ . \end{aligned} \tag{S13}$$

The expression (S13) is identical to Eq. (5) of the main text.

Using the five sets of CR parameters of Fig. 3F-3J, we obtain the values of g_{cr} for the five data sets to be $g_{cr} = g_0 + 0.005$ (for $a = 1.1$), $g_{cr} = g_0$ $(a = 2)$, $g_{cr} = g_0 + 0.001$ $(a = 4)$, $g_{cr} = g_0 + 0.004$ $(a = 9)$, and $g_{cr} = g_0 + 0.08$ $(a = 100)$. When $a \neq 2$ the predicted values of g_{cr} are all slightly higher than $g_0 = (1 + a)/3$, which is the expected payoff per game round for a player adopting the NE mixed strategy. On the empirical side, we compute the mean payoff g_i per game round for each player i in all populations of the same value of a. The mean value of g_i among these players, denoted as $\overline{g}$, is also found to be slightly higher than

g_0 for all the four sets of populations of $a \neq 2$. To be more specific, we observe that $\overline{g} - g_0$ equals to 0.009 ± 0.004 (for $a = 1.1$, mean $\pm$ SEM), 0.000 ± 0.006 ($a = 2$), 0.004 ± 0.012 ($a = 4$), 0.01 ± 0.02 ($a = 9$) and 0.05 ± 0.37 ($a = 100$). These theoretical and empirical results indicate that the conditional response strategy has the potential of bringing higher payoffs to individual players as compared with the NE mixed strategy.

C. The symmetric case

Very surprisingly, we find that asymmetry in the CR parameters is not essential for cycle persistence and direction. We find that if the CR parameters are symmetric with respect to clockwise and counter-clockwise action shifts (namely, $W_+/W_- = T_+/T_- = L_+/L_- = 1$), the cycling frequency f_{cr} is still nonzero as long as $W_0 \neq L_0$. The magnitude of f_{cr} increases with $|W_0 - L_0|$ and decreases with T_0, and the cycling is counter-clockwise ($f_{cr} > 0$) if $W_0 > L_0$ and clockwise ($f_{cr} < 0$) if $L_0 > W_0$, see Supplementary Fig. S3C online. In other words, in this symmetric CR model, if losers are more (less) likely to shift actions than winners, the social state cycling will be counter-clockwise (clockwise).

To give some concrete examples, we symmetrize the transition parameters of Fig. 3F-3J while keeping the empirical values of W_0, T_0, L_0 unchanged. The resulting cycling frequencies are, respectively, $f_{cr} = 0.024$ ($a = 1.1$), 0.017 ($a = 2.0$), 0.017 ($a = 4.0$), 0.015 ($a = 9.0$) and 0.017 ($a = 100.0$), which are all significantly beyond zero. Our model is indeed dramatically different from the best response model, for which asymmetry in decision-making is a basic assumption.

D. Sampling the conditional response parameters

For the population size $N = 6$, we uniformly sample 2.4×10^9 sets of conditional response parameters $W_-, W_0, \ldots, L_0, L_+$ under the constraints of Eq. (S10), and for each of them we determine the theoretical frequency f_{cr} and the theoretical payoff g_{cr} numerically. By this way we obtain the joint probability distribution of f_{cr} and g_{cr} and also the marginal probability distributons of f_{cr} and g_{cr}, see Fig. 4, Supplementary Fig. S1 online and Supplementary Fig. S3A online. The mean values of $|f_{cr}|$ and g_{cr} are then computed from this joint probability distribution. We find that the mean value of f_{cr} is equal to zero, while the

mean value of $|f_{cr}| \approx 0.061$.

The mean value of g_{cr} for randomly sampled CR strategies is determined to be $g_0 - 0.0085(a-2)$ for $N = 6$. When $a > 2$ this mean value is less than g_0, indicating that if the CR parameters are randomly chosen, the CR strategy has high probability of being inferior to the NE mixed strategy.

However, we also notice that g_{cr} can considerably exceed g_0 for some optimized sets of conditional response parameters (see Fig. 4 for the case of $N = 6$ and Supplementary Fig. S1 online for the case of $N = 12$). To give some concrete examples, here we list for population size $N = 6$ the five sets of CR parameters of the highest values of g_{cr} among the sampled 2.4×10^9 sets of parameters:

1. $\{W_- = 0.002, W_0 = 0.998, W_+ = 0.000, T_- = 0.067, T_0 = 0.823, T_+ = 0.110, L_- = 0.003, L_0 = 0.994, L_+ = 0.003\}$. For this set, the cycling frequency is $f_{cr} = 0.003$, and the expected payoff of one game round is $g_{cr} = g_0 + 0.035(a-2)$.

2. $\{W_- = 0.001, W_0 = 0.993, W_+ = 0.006, T_- = 0.154, T_0 = 0.798, T_+ = 0.048, L_- = 0.003, L_0 = 0.994, L_+ = 0.003\}$. For this set, $f_{cr} = 0.007$ and $g_{cr} = g_0 + 0.034(a-2)$.

3. $\{W_- = 0.995, W_0 = 0.004, W_+ = 0.001, T_- = 0.800, T_0 = 0.142, T_+ = 0.058, L_- = 0.988, L_0 = 0.000, L_+ = 0.012\}$. For this set, $f_{cr} = -0.190$ and $g_{cr} = g_0 + 0.034(a-2)$.

4. $\{W_- = 0.001, W_0 = 0.994, W_+ = 0.004, T_- = 0.063, T_0 = 0.146, T_+ = 0.791, L_- = 0.989, L_0 = 0.010, L_+ = 0.001\}$. For this set, $f_{cr} = 0.189$ and $g_{cr} = g_0 + 0.033(a-2)$.

5. $\{W_- = 0.001, W_0 = 0.992, W_+ = 0.006, T_- = 0.167, T_0 = 0.080, T_+ = 0.753, L_- = 0.998, L_0 = 0.000, L_+ = 0.002\}$. For this set, $f_{cr} = 0.179$ and $g_{cr} = g_0 + 0.033(a-2)$.

To determine the influence of each of the nine conditional response parameters to the cycling frequency f_{cr}, we fix each of these nine conditional response parameters and sample all the others uniformly at random under the constraints of Eq. (S10). The mean value $\langle f_{cr} \rangle$ of f_{cr} as a function of this fixed conditional response parameter is then obtained by repeating this process many times, see Supplementary Fig. S3B online. As expected, we find that when the fixed conditional response parameter is equal to 1/3, the mean cycling frequency $\langle f_{cr} \rangle = 0$. Furthermore we find that

1. If W_0, T_+ or L_+ is the fixed parameter, then $\langle f_{cr} \rangle$ increases (almost linearly) with fixed parameter, indicating that a larger value of W_0, T_+ or L_+ promotes counter-clockwise cycling at the population level.

2. If W_-, T_- or L_0 is the fixed parameter, then $\langle f_{cr} \rangle$ decreases (almost linearly) with this fixed parameter, indicating that a larger value of W_-, T_- or L_0 promotes clockwise cycling at the population level.

3. If W_+, T_0 or L_- is the fixed parameter, then $\langle f_{cr} \rangle$ does not change with this fixed parameter (i.e., $\langle f_{cr} \rangle = 0$), indicating that these three conditional response parameters are neutral as the cycling direction is concerned.

E. Action marginal distribution of a single player

The social state transition matrix Eq. (8) has the following rotation symmetry:

$$\begin{aligned} M_{cr}[(n'_R, n'_P, n'_S)|(n_R, n_P, n_S)] &= M_{cr}[(n'_S, n'_R, n'_P)|(n_S, n_R, n_P)] \\ &= M_{cr}[(n'_P, n'_S, n'_R)|(n_P, n_S, n_R)] \ . \end{aligned} \tag{S14}$$

Because of this rotation symmetry, the steady-state distribution $P^*_{cr}(\mathbf{s})$ has also the rotation symmetry that

$$P^*_{cr}(n_R, n_P, n_S) = P^*_{cr}(n_S, n_R, n_P) = P^*_{cr}(n_P, n_S, n_R) \ . \tag{S15}$$

After the social states of the population has reached the steady-state distribution $P^*_{cr}(\mathbf{s})$, the probability ρ^R_{cr} that a randomly chosen player adopts action R in one game round is expressed as

$$\rho^R_{cr} = \sum_{\mathbf{s}} P^*_{cr}(\mathbf{s}) \frac{n_R}{n_R + n_P + n_S} = \frac{1}{N} \sum_{\mathbf{s}} P^*_{cr}(\mathbf{s}) n_R \ , \tag{S16}$$

where the summation is over all the possible social states $\mathbf{s} = (n_R, n_P, n_S)$. The probabilities ρ^P_{cr} and ρ^S_{cr} that a randomly chosen player adopts action P and S in one play can be computed similarly. Because of the rotation symmetry Eq. (S15) of $P^*_{cr}(\mathbf{s})$, we obtain that $\rho^R_{cr} = \rho^P_{cr} = \rho^S_{cr} = 1/3$.

Therefore, if the players of the population all play the same CR strategy, then after

the population reaches the the steady-state, the action marginal distribution of each player will be identical to the NE mixed strategy. In other words, the CR strategy can not be distinguished from the NE mixed strategy through measure the action marginal distributions of individual players.

F. Computer simulations

All of the theoretical predictions of the CR model have been confirmed by extensive computer simulations. In each of our computer simulation processes, a population of N players repeatedly play the RPS game under the random pairwise-matching protocol. At each game round, each player of this population makes a choice on her action following exactly the CR strategy. The parameters $\{W_-, W_0, \ldots, L_+\}$ of this strategy is specified at the start of the simulation and they do not change during the simulation process.

S5. THE GENERALIZED CONDITIONAL RESPONSE MODEL

If the payoff matrix of the RPS model is more complex than the one shown in Fig. 1A, the conditional response model may still be applicable after some appropriate extensions. In the most general case, we can assume that a player's decision is influenced by the player's own action and the opponent's action in the previous game round.

Let us denote by $q_s \in \{R, P, S\}$ a player's action at time t, and by $q_o \in \{R, P, S\}$ the action of this player's opponent at time t. Then at time $(t+1)$, the probability that this player adopts action $q \in \{R, P, S\}$ is denoted as $Q^q_{(q_s, q_o)}$, with the normalization condition that

$$Q^R_{(q_s,q_o)} + Q^P_{(q_s,q_o)} + Q^S_{(q_s,q_o)} \equiv 1 \ . \tag{S17}$$

This generalized conditional response model has 27 transition parameters, which are constrained by 9 normalization conditions [see Eq. (S17)]. The social state transition matrix of this generalized model is slightly more complicated than Eq. (8).

The win-lose-tie conditional response model is a limiting case of this more general model. It can be derived from this general model by assuming $Q^q_{(R,R)} = Q^q_{(P,P)} = Q^q_{(S,S)}$, $Q^q_{(R,P)} = Q^q_{(P,S)} = Q^q_{(S,R)}$, and $Q^q_{(R,S)} = Q^q_{(P,R)} = Q^q_{(S,P)}$. These additional assumptions are reasonable only for the simplest payoff matrix shown in Fig. 1A.
